# In vivo and ex vivo effects of propofol on myocardial performance in rats with obstructive jaundice

**DOI:** 10.1186/1471-230X-11-144

**Published:** 2011-12-28

**Authors:** Hong-Mei Ren, Li-Qun Yang, Zhi-Qiang Liu, Cai-Yang Chen, Chi-Wai Cheung, Kun-Ming Tao, Jian-Gang Song, Wu-Rong Chen, Wei-Feng Yu

**Affiliations:** 1 Department of Anesthesia, Putuo Hospital, Shanghai, China; 2 Department of Anesthesia and Intensive Care, Eastern Hepatobiliary Surgical Hospital, the Second Military Medical University, Shanghai, China; 3 Department of Anesthesiology, Shanghai First Maternity and Infant Hospital, Tongji University School of Medicine, Shanghai, China; 4 Department of Anaesthesiology, University of Hong Kong, Hong Kong, China

## Abstract

**Background:**

Responsiveness of the "jaundiced heart" to propofol is not completely understood. The purpose of this study was to evaluate the effect of propofol on myocardial performance in rats with obstructive jaundice.

**Methods:**

Male Sprague-Dawley rats (n = 40) were randomly allocated into two groups, twenty underwent bile duct ligation (BDL), and 20 underwent a sham operation. Seven days after the surgery, propofol was administered in vivo and ex vivo (Langendorff preparations). Heart rate, left ventricular end-systolic pressure (LVESP) left ventricular end-diastolic pressure (LVEDP), and maximal rate for left ventricular pressure rise and decline (± dP/dt_max _) were measured to determine the influence of propofol on the cardiac function of rats.

**Results:**

Impaired basal cardiac function was observed in the isolated BDL hearts, whereas in vivo indices of basal cardiac function (LVESP and ± dP/dt) in vivo were significantly higher in rats that underwent BDL compared with controls. With low or intermediate concentrations of propofol, these indices of cardiac function were within the normal physiologic range in both groups, and responsiveness to propofol was unaffected by BDL. When the highest concentration of propofol was administrated, a significant decline in cardiac function was observed in the BDL group.

**Conclusions:**

In rats that underwent BDL, basal cardiac performance was better in vivo and worse ex vivo compared with controls. Low and intermediate concentrations of propofol did not appear to impair cardiac function in rats with obstructive jaundice.

## Background

Postoperative obstructive jaundice is associated with multiple organ dysfunction syndrome [1-3]. Specifically, the cardiovascular instability caused by defects in myocardial performance and vascular reactivity is thought to be an important mechanism in the pathophysiology of multiple organ dysfunction syndrome [4,5]. In 1986, Green [5] reported impaired left ventricular performance in dogs with cholemia, and called this hepatic cardiomyopathy "jaundiced heart"[4]. The jaundiced heart is also characterized by defective vascular reactivity attributed to altered beta-adrenergic receptor signaling [6], membrane fluidity, and down regulation of cardiac beta-adrenoceptor density and affinity [7].

Propofol is a widely employed intravenous anesthetic used to induce and maintain anesthesia. It is characterized by rapid onset/offset of effect and rapid elimination from the body [8,9]. However, propofol can also induce cardiovascular depression, manifested primarily by decreased arterial blood pressure [10] due to inhibition of the sympathetic nervous system [11], a negative inotropic effect [12], and reduced preload [13].

The effect of propofol on myocardial performance in patients with obstructive jaundice is unclear. Therefore, in the present study, 7-day bile duct ligation (BDL) was used as a model of obstructive jaundice to evaluate the effect of propofol on cardiac function in vivo. In ex vivo experiments, the direct effects of propofol on the isolated heart of BDL rats were assessed to exclude the vasodilator effect of propofol and the associated neurohormonal adaptation in vivo [14,15].

## Methods

### Animals

This study protocol was approved by the Institutional Animal Care and Use Committee at the Second Military Medical University. Male Sprague-Dawley rats (250-300 g) were housed in a temperature and humidity-controlled environment with a 12-h light dark cycle. The rats received unlimited access to water and food. Food was removed from the cage 16 hours before initiating the experiments.

### Procedures

Forty rats were randomly allocated into two study groups: 20 underwent the BDL procedure, and 20 underwent a sham operation (control), according to Lee [16]. Rats were anesthetized with pentobarbital sodium (40 mg/kg by intraperitoneal [i.p.] administration). After opening the abdominal cavity, the bile duct was ligated at a point proximal to the hilus and a point immediately distal to the entry of the bile duct into the duodenum. The bile duct was then severed between the ligatures. The sham operation was carried out in a similar manner, but did not include ligating and severing the bile duct. Seven days after the BDL and sham procedures, blood was obtained to assess total bilirubin and alanine aminotransferase levels.

### In vivo experiment

For the in vivo experiments, all surgical procedures were performed under sterile conditions. On the seventh postoperative day, rats (BDL group, *n *= 10; sham-operated control group, *n *= 10) were anesthetized with sodium pentobarbital (40 mg/kg i.p.). After tracheotomy and tracheal intubation, mechanical ventilation (room air at 60-70 breaths/min) was provided with a Harvard Apparatus Rodent Respirator (Harvard Apparatus, Boston, MA). A heating pad was placed under the animal to keep the rectal temperature at 36°C ± 1°C. A 1-cm midline incision was made in the neck, and the right common carotid artery was exposed. A polyethylene cannula was inserted through the artery to the left ventricle. To monitor intraventricular pressure, the cannula was connected to a pressure transducer (RM6240B type; Chengdu Instrument Corp., China). A cannula connected to a microinfusion pump (Graseby 3500; Graseby Medical Ltd., Watford, UK) via a three-port switch was placed in the left jugular vein for propofol administration. After a 30-min stabilization period, rats received an intravenous injection of propofol (Diprivan 2%, AstraZeneca UK Ltd UK) The dose range used in our study encompassed the 50% effective dose for propofol as reported by Carmichael et al. [17]. Doses of 300, 600 and 900 μg/kg per minute of propofol were administered by infusion (15 min each dose) to generate dose-response curves for heart rate (HR), left ventricular end-systolic pressure (LVESP), and maximal rate for left ventricular pressure rise and declining (± dP/dt_max _). HR and cardiac function indices were determined at the point of maximum response for each propofol dose.

### Ex vivo experiment

Langendorff heart preparation (ML870B2 AD Instruments Ltd, Shanghai, China) was used to evaluate the effects of propofol on cardiac performance. The rats (BDL group, *n *= 10; sham-operated control group, *n *= 10) were anesthetized with sodium pentobarbital (40 mg/kg i.p.). The heart was removed from the thoracic cavity and placed in ice-cold Krebs-Henseleit bicarbonate buffer (pH 7.4) containing heparin sodium. The heart was rapidly mounted on a Langendorff perfusion system via the aorta. A cardiac catheter, with a latex saccule at the tip, was inserted from a cut in the left auricle of the left atrium to the left ventricle. The saccule was filled with water to maintain the left ventricular end-diastolic pressure (LVEDP) at 6 to 8 mmHg. The other end of the catheter was connected to a pressure transducer (MLT844; Shanghai, China). The average surrounding pressure was 70 to 80 mmHg. The perfusion medium was a modified Krebs-Henseleit bicarbonate buffer gassed with 95% O2 and 5% CO_2 _at atmospheric pressure and maintained at 37.5°C. The buffer was composed of 118 mmol/L NaCl, 4.7 mmol/L KCl, 1.2 mmol/L KH_2 _PO_4 _, 25 mmol/L NaHCO_3 _, 2.5 mmol/L CaCl_2 _, 11.0 mmol/L glucose, 0.5 mmol/L EDTA-Na_2 _and 1.2 mmol/L MgSO_4 _. Recorded parameters included HR, LVESP, LVEDP, (and ± dP/dt_max _). Effects of propofol (12.5, 25, and 50 μmol/L) were tested using a method described by Jacob et al [18]. In rats that underwent 3-day BDL, Jacob [18] showed impaired basal cardiac contractility and reduced left intraventricular pressure using two preparations of pithed rats and isolated functioning hearts. When the aortic flow and left ventricular pressure were stabilized, data were acquired for 30 consecutive seconds prior to drug testing. Each drug concentration was tested for a period of 15 min.

### Statistical methods

Data analysis was performed with Prism version 4.0 (GraphPad Software; San Diego, CA). Responses of cardiac function to propofol are expressed as mean ± standard error of the mean (SEM) relative to baseline values (percentage). Group results were compared by Student's unpaired t-test or the chi-square test. In vivo and ex vivo myocardial performance data were compared by two-way repeated measure analysis of variance (ANOVA) followed by the post hoc Bonferroni test. A P-value of < 0.05 was considered significant.

## Results

Serum total bilirubin and alanine transaminase in the BDL group were significantly higher than those in the sham-operated control group (total bilirubin, 1.4 ± 0.2 *vs *158.7 ± 10.8 mM/L, respectively; alanine transaminase, 28.1 ± 1.9 *vs *150.0 ± 14.2 IU/L respectively)(P < 0.01).

### In vivo cardiac function

**Figure 1:**
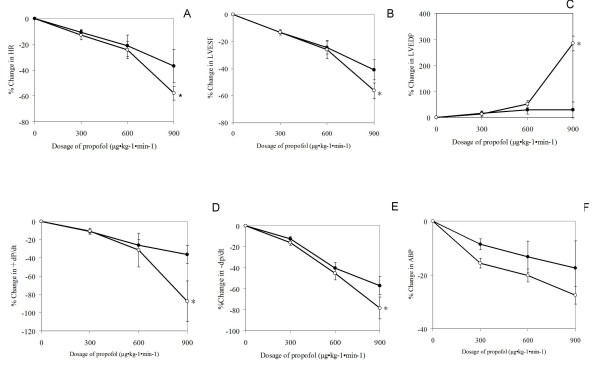
**In vivo effect of propofol on heart rate and indices of cardiac contractility on day 7 in a rat model of obstructive jaundiced and sham-operated controls. (A) **Heart rates. **(B) **Left ventricular end-systolic pressure (LVESP); **(C) **Left ventricular end-diastolic pressure (LVEDP); **(D) **Maximal rate for left ventricular pressure rise (+dT/dt_max _). **(E) **Maximal rate of left ventricular pressure declining (-dP/dt_max _). **(F) **Arterial blood pressure. Results are expressed as mean ± SEM(*n *= 10). SO, sham-operated control; BDL, bile duct ligation; HR, heart rate; Abscissa, propofol concentration; ordinate, percent change in a given variable compared with baseline; closed circle, sham-operated rat; open circle, BDL rat. For each twitch parameter, original hemodynamic data from the experimental groups were compared by two-way ANOVA followed by the Bonferroni test. * P < 0.05 *vs *sham-operated controls (same propofol concentration).

The baseline contractility and lusitropy (LVESP and ± dP/dt_max _) of the BDL group were higher than those of the sham-operated controls (P < 0.05), although LVEDP did not differ significantly between the two study groups (Table 1). Blood pressure decreased in the BDL group after propofol administration but there was no significant difference compared with blood pressure after the same propofol concentrations in the control group. At an infusion rate of 300 to 600 μg/kg per min, propofol decreased HR, LVESP, and ± dP/dt_max _, but not LVEDP. The decreases in HR, LVESP, and ± dP/dt_max _were dose-dependent in both experimental groups(Figure 1). At 900 μg/kg per min, the inhibitory effects of propofol on cardiac performance were greater in the BDL group than in the sham-operated control group (P < 0.05)(Figure 1).

**Table 1:** In vivo baseline heart rate and cardiac function in the BDL group and sham control group

	HR (bpm)	LVESP (mmHg)	LVEDP (mmHg)	**+dP/dt**_max _**(mmHg s^-1^)**	-**dP/dt**_max _**(mmHg s^-1^)**	ABP (mmHg)
SO	355.5 ± 12.7	110.1 ± 4.3	3.7 ± 0.4	2173.6 ± 82.3	1249.7 ± 92.6	105 ± 17
BDL	311.3 ± 16.7*	127.2 ± 6.4*	4.5 ± 0.5	3288.7 ± 112.3*	2512.8 ± 132.5*	109 ± 13

(*n *= 10 in each group)

Values are means ± SEM; *n*, sample size; SO, sham-operated; BDL, bile duct ligation; HR, heart rate; LVESP, left ventricular end-systolic pressure; LVEDP, left ventricular end-diastolic pressure; ± dP/dt_max _, maximal rate for left ventricular pressure rising and declining; ABP, arterial blood pressure.

* P < 0.05 *vs *sham controls.

### Ex vivo cardiac function

**Figure 2:**
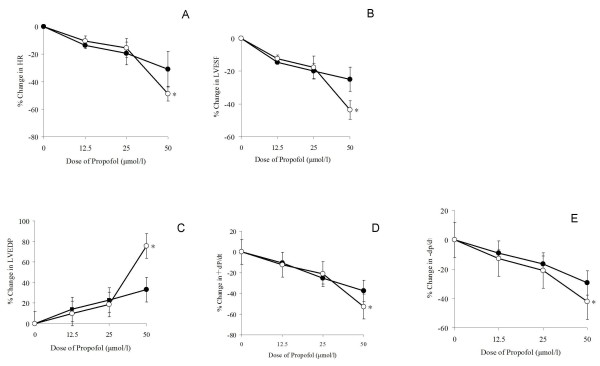
**Effect of propofol on heart rates and indices of cardiac contractility ex vivo (Langendorff preparations) at day 7 in a rat model of obstructive jaundice and sham-operated controls. (A) **Heart rates. **(B) **Maximal rate of left ventricular pressure rise (+dT/dt). **(C) **Left ventricular end-systolic pressure (LVESP); **(D) **Left ventricular end-diastolic pressure (LVEDP). **(E) **Maximal rate of left ventricular pressure declining (-dP/dt_max _). Results are expressed as means ± SEM (*n *= 10); SO, sham-operated control; BDL, bile duct ligation; HR, heart rate; Abscissa, propofol concentrations; ordinate, percent change in a given variable compared with baseline; closed circle, sham-operated control; open circle, BDL group. For each twitch parameter, original hemodynamic data from the groups were compared by two-way ANOVA followed by the Bonferroni test. * P < 0.05 *vs *sham-operated controls (same propofol concentration).

At baseline, LVESP, ± dP/dt, LVEDP and HR were significantly lower in the BDL group than in the control group (P < 0.05)(table 2). At 12.5 and 50 μmol/L, propofol significantly decreased HR, LVESP, and ± dP/dt_max _, but increased LVEDP. The dose-dependent decrease in response appeared to be more pronounced in the BDL group, and this difference was significant at 50 μmol/L of propofol (P < 0.01)(Figure 2).

**Table 2:** Ex vivo baseline heart rate and cardiac function in the BDL group and the sham control group

	HR (bpm)	LVESP (mmHg)	LVEDP (mmHg)	+dP/dt_max _(mmHg s-1)	-dP/dt_max _(mmHg s-1)
SO (n = 10)	254.5 ± 19.5	112.5 ± 6.9	4.8 ± 0.5	1469.2 ± 157.4	1117.5 ± 70.0
BDL (n = 10)	201.3 ± 13.5*	92.4 ± 5.4*	7.4 ± 0.3*	1146.0 ± 64.2*	954.3 ± 103.7*

Values are means ± SEM; *n*, sample size; SO, sham operated; BDL, bile duct ligation; HR, heart rate; LVESP, left ventricular end-systolic pressure; LVEDP, left ventricular end-diastolic pressure; ± dP/dt_max _, maximal rate for left ventricular pressure rising and declining.

* P < 0.05 *vs *sham controls.

## Discussion

In the present study, we could not confirm impaired cardiac performance in a rat model of obstructive jaundice because the in vivo and ex vivo results were inconsistent. However, an important and novel finding is that the inhibitory effects of propofol on myocardial function were comparable in rats that underwent BDL and rats that underwent the sham operation at low and intermediate doses (12.5 and 25 μmol/L). At the highest dose of propofol tested (50 μmol/L), stronger effects on cardiac function were observed in rats with obstructive jaundice.

A propofol concentration of 2 to 4 μM was clinically relevant according to a previous study [19], and researchers proved that higher than > 10 μM propofol in vitro might cause cardiovascular depression [20]. To compare the hemodynamic effects of propofol between cholestatic rat hearts and controls, we use d relatively high concentrations (12.5, 25, and 50 μmol/L) of propofol ex vivo because propofol at < 10 μM did not suppress the KATP channel or display a myocardial depression effect [21]. The present study did not confirm our hypothesis that propofol depresses myocardial performance in the jaundiced heart compared with the normal, healthy heart. This finding may be attributed to a number of factors. First, most animal and human studies have shown that propofol induces dose-dependent cardiovascular depression. Furthermore, clinically relevant concentrations of propofol do not significantly depress myocardial contractility and evidence suggests that its pharmacological properties may be due in part to enhanced sensitivity of the myofilaments to Ca^2+^, despite reduced uptake of Ca^2+ ^uptake into the sarcoplasmic reticulum [22,23]. Moreover, propofol is a cardioprotective agent and may protect the myocardium from injury such as that caused by ischemia-reperfusion [24,25]. Inhibition of the mitochondrial permeability transition pore is a recently reported mechanism underlying the protective action of propofol on the myocardium [24,26]. In addition, accumulation of bile acids is a causative factor for jaundiced heart [5]. Bile acids exert negative chronotropic and inotropic effects on the heart via mitochondrial damage [12,27]. These factors especially the enhanced sensitivity of the myofilaments to Ca^2+ ^and the inhibition of the mitochondrial permeability transition pore, may protect against cardiovascular depression and account for the minimal responsiveness of BDL-treated rats to low and intermediate concentrations of propofol.

Another interesting observation was the opposite in vivo and ex vivo results of basal cardiac contractility in the BDL group. In the ex vivo experiments, hearts from the BDL-treated rats exhibited impaired contractility and a reduction in the maximum pressure in the left ventricle, whereas BDL-treated rats showed significantly increased LVSEP, +dP/dt_max _and -dP/dt_max _in vivo. The mechanism underlying this phenomenon is unclear, but may involve neural or humoral adaptations. Dabagh [28] reported increased concentrations of norepinephrine and epinephrine during the development of cholestasis. Poo [29] found elevated renin and angiotensin II at 1 week after establishment of obstructive jaundice in rats. In our in vivo experiments, increased LVSEP, +dP/dt_max _and -dP/dt_max _, were associated with myocardial contractility, suggesting up regulation of the sympathetic nervous system. In addition, LVEDP which directly relates to the cardiac preload might remain unchanged possibly not owing to the depletion of body fluid in obstructive jaundice [30,31]. However, the lower heart rate may be caused in part by accumulated bile acids, which impair cardiac function by affecting calcium uptake from the sarcoplasmic reticulum [32,33]. Recent research has focused on the overproduction of nitric oxide, which results in bradycardia after BDL [34,35]. Despite enhanced cardiac contractility in vivo, our experiment showed that a high dose of propofol produced stronger inhibition of cardiac function in BDL-treated rats, unmasking latent cardiac dysfunction. These results are consistent with those of previous studies reporting that BDL-treated rats show hemodynamic instability [5].

There are also limitations in our study. To assess left ventricular inotropism, three load-dependent indices of left ventricular contractility (LVESP, LVEDP, and ± dP/dt_max _) were used. It must be underscored that these indices may also be influenced by left ventricular loading conditions [36]. Load-independent indices of left ventricular contractility, such as construction of pressure-volume curves in both systole and diastole would greatly enhance the validity and interpretation of our results. It would enable more specific measurement of the left ventricular performance independent of both loading conditions and HR [37]. Thus, the data of our study must be interpreted with caution when using propofol at mild to intermediate doses in obstructive jaundice.

## Conclusions

In conclusion, the present study demonstrates that obstructive jaundice impairs cardiac function ex vivo but enhances cardiac performance in vivo. Propofol depresses cardiac parameters at low and intermediate doses to a similar degree in BDL-treated and sham-operated rats. However, at a high dose, propofol may cause exaggerated cardiac depression in jaundiced rats. Our study suggestes that propofol is a safe alternative anesthetic agent in patients with obstructive jaundice and normal cardiac function at low and intermediate doses.

## Competing interests

The authors declare that they have no competing interests.

## Authors' contributions

HR: data mining and analysis in fulfillment of his MD thesis. LY and ZL: idea, experimental design and part of animal studies. CC and KT: conception and help with statistics. CC: conception writing of the paper. JS and WC: ex vivo experiment. WY: idea, experimental design and part of animal studies. All authors read and approved the final version of the manuscript.

## Pre-publication history

The pre-publication history for this paper can be accessed here:

http://www.biomedcentral.com/1471-230X/11/144/prepub
